# GC-Content of Synonymous Codons Profoundly Influences Amino Acid Usage

**DOI:** 10.1534/g3.115.019877

**Published:** 2015-08-06

**Authors:** Jing Li, Jun Zhou, Ying Wu, Sihai Yang, Dacheng Tian

**Affiliations:** * State Key Laboratory of Pharmaceutical Biotechnology, School of Life Sciences, Nanjing University, Nanjing 210093, China; † Institute for Research on Cancer and Aging, Nice (IRCAN), Nice, France; ‡ Department of Organismic and Evolutionary Biology, Harvard University, Cambridge, Massachusetts; § Institute for Bioscience and Biotechnology Research, University of Maryland, Rockville, Maryland

**Keywords:** GC-content, synonymous codon, amino acid usage, codon usage

## Abstract

Amino acids typically are encoded by multiple synonymous codons that are not used with the same frequency. Codon usage bias has drawn considerable attention, and several explanations have been offered, including variation in GC-content between species. Focusing on a simple parameter—combined GC proportion of all the synonymous codons for a particular amino acid, termed GC_syn_—we try to deepen our understanding of the relationship between GC-content and amino acid/codon usage in more details. We analyzed 65 widely distributed representative species and found a close association between GC_syn_, GC-content, and amino acids usage. The overall usages of the four amino acids with the greatest GC_syn_ and the five amino acids with the lowest GC_syn_ both vary with the regional GC-content, whereas the usage of the remaining 11 amino acids with intermediate GC_syn_ is less variable. More interesting, we discovered that codon usage frequencies are nearly constant in regions with similar GC-content. We further quantified the effects of regional GC-content variation (low to high) on amino acid usage and found that GC-content determines the usage variation of amino acids, especially those with extremely high GC_syn_, which accounts for 76.7% of the changed GC-content for those regions. Our results suggest that GC_syn_ correlates with GC-content and has impact on codon/amino acid usage. These findings suggest a novel approach to understanding the role of codon and amino acid usage in shaping genomic architecture and evolutionary patterns of organisms.

The genetic code describes how the 64-nucleotide triplets specify 20 amino acids. Most amino acids have at least two synonymous codons that are, however, not used at the same frequencies in different genomes. Grantham *et al.* (1980) proposed the “genome hypothesis” in 1980 that assumed a species-specific pattern of codon usage. Interestingly, even in the same genome, the codon usage varies significantly among genes with different expression levels (Dos Reis *et al.* 2003), functions (Chiapello *et al.* 1998; Karlin *et al.* 1998; Liu *et al.* 2005), and tissue-specific patterns (Plotkin *et al.* 2004). Various factors have been suggested to affect codon usage bias, such as relative abundance of iso-accepting transfer RNAs, gene expression level, gene length, gene conversion, messenger RNA structure, and DNA base composition (Miyata *et al.* 1979; Ikemura 1981; Gouy and Gautier 1982; Sharp *et al.* 1986; Eyre-Walker 1996; Duret and Mouchiroud 1999; Sueoka and Kawanishi 2000; Maside *et al.* 2004). The most significant factor linked to the phenomenon of codon bias between different organisms is perhaps GC-content.

The great influence of GC-content on codon bias was first predicted by Sueoka in the 1960s (Sueoka 1961, 1962). With limited available nucleotide sequences during the 1980s and 1990s, intragenomic comparisons of heterologous DNA and protein sequences (Bernardi and Bernardi 1986; D’Onofrio *et al.* 1991; Collins and Jukes 1993; Berkhout and Van Hemert 1994; Porter 1995) and intergenomic comparisons of homologous gene sequences (Lobry 1997; Gu *et al.* 1998; Wilquet and Van De Casteele 1999; Lafay *et al.* 1999; D’Onofrio *et al.* 1999) were performed to confirm the correlations between the nucleotide composition of DNA and the amino acid content of the encoded proteins. Later, when more sequenced genomes became available, Knight *et al.* (2001) found similar results and suggested that GC-content is the drive for codon usage and that the correlation between GC-content and amino acid or codon usage is modulated by both mutation and selection. Another study showed that the genome-wide codon bias in eubacteria and archaea could be predicted from intergenic sequences that are not translated, suggesting that genome-wide codon bias is determined primarily by mutational processes throughout the genome (Chen *et al.* 2004). On the basis of the complete genome sequences, Singer and Hickey (2000) partitioned the universal codon table into GC-rich, AT-rich, and neutral codons. They further confirmed a prediction that GC-rich coding sequences (CDS) would encode amino acids with GC-rich codons, showing that biased DNA encodes biased proteins on a genome-wide scale. The finding that a positive correlation between the degree of amino acid bias and the magnitude of protein sequence divergence further support that mutational bias can have a major effect on the molecular evolution of proteins (Singer and Hickey 2000). The influence of GC-content to codon bias also was demonstrated by other studies (Karlin and Mrázek 1996; Kudla *et al.* 2006; Hildebrand *et al.* 2010; Nabiyouni *et al.* 2013; Bohlin *et al.* 2013).

Mutation and natural selection are suggested to be the two main forces shaping the genomic codon and amino acid usage patterns within and between species (Duret 2002; Chamary *et al.* 2006; Hershberg and Petrov 2008). The mutational explanation posits that codon bias arises from biases in nucleotides composition that are produced by point mutations, contextual biases in the point mutation rates or biases in repair. It is neutral without any fitness advantages. In contrast, the natural selection explanation suggests that synonymous mutations would influence the fitness of organisms and therefore be promoted or repressed during evolution. Despite the fact that both remain elusive, these two mechanisms are not mutually exclusive but can both play important roles in patterning the codon and amino acid usage in genomes (Bulmer 1991; Duret 2002; Hershberg and Petrov 2008).

Even though codon usage bias has been documented extensively (Karlin *et al.* 1998; Chen *et al.* 2004; Gustafsson *et al.* 2004; Liu *et al.* 2005; Hershberg and Petrov 2008), any further understanding of codon usage would still have important implications for molecular and genomic evolution. Here, we intend to investigate the impacts of GC-content on codon/amino acid usage quantitatively in more detail, starting from and focusing on a simple parameter, GC proportion of all the synonymous codons for a particular amino acid. We term this proportion the GC_syn_. In this way, the GC-content of codons is defined quantitatively rather than qualitatively regarded as GC-rich or AT-rich. On the basis of GC_syn_, the 20 amino acids are distinctly classified into three groups (four high-, 11 intermediate-, and five low-GC_syn_ amino acids), and their usage characteristics could be analyzed independently. Using 65 representative genomes from diverged species including bacteria, plants, and animals, we identified different associations between usage pattern and synonymous codon numbers for an amino acid from different GC_syn_ groups, indicative of adaptive evolution. More important, according to distinct usage patterns of the three groups of amino acids, we audaciously predict that identical codon/amino acid usage patterns exist when GC-content is similar, regardless of species or lineage. Comprehensive investigation of codon usage using three different units—50 consecutive codons, CDS, and genomes—in diverse prokaryotic and eukaryotic organisms indicates that our predictions are true, indicating that GC-content is a pronounced determinant of amino acid usage. In addition, we created an equation to estimate the degree of GC-content determinant to amino acid composition and GC_syn_ variation in different regions of the genomes. Overall, our results provide a novel view to understand codon bias and its role in the formation of genome architecture.

## Materials and Methods

### Selection of genome sequences

Fifteen eukaryotic and 50 prokaryotic genomes were selected on the basis of the following criteria: widely represented eukaryotic genomes according to phylogeny, and all the prokaryotic genomes greater than 4 Mb in the National Center for Biotechnology Information (ftp://ftp.ncbi.nlm.nih.gov). The genomes and annotations of animals were retrieved from the Ensembl database (www.ensembl.org), and those of *Arabidopsis thaliana* and *Oryza sativa* were downloaded from TIGR (http://www.tigr.org) and GRAMENE (http://www.gramene.org), respectively. The genomic information of other plants was obtained from JGI (ftp://ftp.jgi-psf.org/pub/JGI_data/). The CDS of each species were extracted with the annotations from corresponding, aforementioned Web sites by the use of Perl programs. Protein coding genes with ambiguous bases were eliminated. For the genes with multitranscripts, we chose the longest CDS.

### Classification of the analysis units

**Figure 1:**
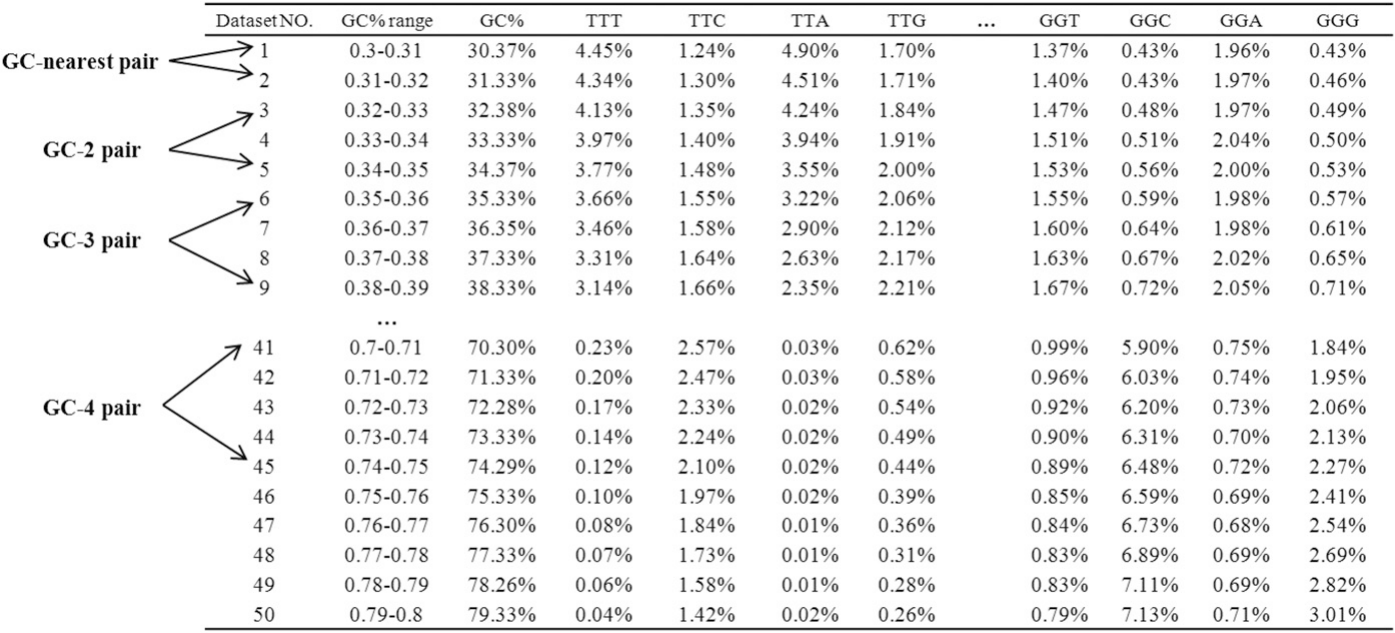
The schematic diagram of codon usage analysis. As is shown in the figure, the table represents some of the codon usage results of 50-consecutive codon group and the table was sorted by the GC-content range of each group. The first column is the number of each dataset line; the second column lists the GC-content range of each 50 consecutive codon group; the third column is the average GC-content of each group; and the remaining columns show the usage frequencies of each synonymous codon. GC-nearest pair consists of two datasets with the nearest GC-content (*i.e.*, the nearest dataset line number, such as datasets 1 and 2, 3 and 4, …, 49 and 50 in the table); GC-N pair consists of two datasets with larger GC-content divergence and N is the discrepancy between the number of any two datasets (*e.g.*, GC-2 pairs contain the pairs of datasets 1 and 3, 2 and 4, 3 and 5…48 and 50; GC-3 pairs contain the pairs of datasets 1 and 4, 2 and 5, 3 and 6 … 47 and 50).

To investigate the correlation between the frequency of amino acid (or codon) usage and GC-content, we used the whole-genome sequences of different species, CDS, or 50 consecutive codons as separate units. Of note, we also tested 30 consecutive codons and 100 consecutive codons as analysis units in comparison with 50 consecutive codons analyses. The results were highly similar so that we focused on the 50 consecutive codons analysis in the following work. For the “whole-genome sequence” analyze unit, the organisms were ranked according to their genomic GC-content from low to high. For the “CDS” analyze unit, we combined CDSs from 65 organisms and sorted them based on their GC-content, which ranged from 0.30 to 0.80, into 50 groups with an equal interval of 0.01 (second column of Figure 1). Therefore, every group of CDS contains sequences from multiple species instead of from only one genome, which eliminates potential genome biases since any genome may have a particular coding strategy as suggested in Grantham’s genome hypothesis (Grantham *et al.* 1980). For the “50 consecutive codon” units, each CDS in any genome was dissected sequentially into sets of 50 consecutive codons, whereas the remaining regions shorter than 50 codons were discarded. This largely diminishes the influence of special or functional motifs in a gene. These sequences also were sorted into 50 groups in the same way as the CDS with a GC interval of 0.01. Since the fractions of sequences whose GC-content is <0.3 or >0.8 were small (total < 1.30%), they were excluded from further analyses.

### Linear regression analysis of codon usage

We calculated the usage of all the 61 codons (excluding the start and stop codons; of note, we excluded the first Met encoded by start codon in CDS, which would lead to biased analysis of amino acid usage) in all the 65 genomes that were first orderly ranked based on genomic GC-content. Similarly, for “CDS” and “50 consecutive codon” unit, we acquired codon usage data for each of the 50 groups (Figure 1 depicts the organization of the data). Then, we compared the codon usage in any possible pairs, such as two organisms, two groups of CDSs, and two groups of 50 consecutive codons segments. The codon usage data of two groups (*i.e.*, to compare the linear regression correlation between two sets of the individual codon usage data as demonstrated in the last column of Figure 1) were plotted on x- and y-axes, respectively. More specifically, to analyze the group pairs with the nearest GC-content, we used the sorted codon usage tables and performed the linear regression analysis on the adjacent group pairs to get the slope and correlation coefficient (Figure 1 “GC-nearest pair”). GC-nearest pair consists of two datasets with the nearest GC-content (*i.e.*, the nearest dataset line number, such as datasets 1 and 2, 3 and 4, …, 49 and 50 in the table). In addition, we also conducted regression analysis between groups with greater GC-content divergence and calculated slope and R^2^, which were defined as GC-2 pair, GC-3 pair... GC-N pair (Figure 1). GC-N pair consists of two datasets with larger GC-content divergence and N is the discrepancy between the number of any two datasets (*e.g.*, GC-2 pairs contain the pairs of datasets 1 and 3, 2 and 4, 3 and 5…48 and 50; GC-3 pairs contain the pairs of datasets 1 and 4, 2 and 5, 3 and 6 … 47 and 50). The average values of the slope and R^2^ between GC-nearest pairs, GC-2 pairs, GC-3 pairs…GC-N pairs were calculated and plotted against the difference of GC-content.

The linear regression analysis was conducted in the software package R (version 2.14.1) to derive the correlation coefficient (R^2^) and the slope of the linear correlation between pairs. For every paired comparison, regression analyses were conducted for the usages of both 20 amino acids and 61 sense codons.

### Estimation of the effects of regional GC-content

To estimate the effects of increased regional GC-content (*i.e.*, ΔGC, the difference of GC-content between the regions with 0.79−0.80 and 0.30−0.31 GC-content), we split it into two components, which are (1) the GC fraction change due to the use of different amino acids and (2) the GC change due to the use of different synonymous codons for the same amino acid. The following formula was used:

$$\begin{array}{c} \Delta \text{GC}={\text{A}}_{0.8}\times {\text{GC}}_{\text{syn}0.8}-{\text{A}}_{0.3}\times {\text{GC}}_{\text{syn}0.3}\\ ={\text{A}}_{0.8}\times {\text{GC}}_{\text{syn}0.8}-{\text{A}}_{0.3}\times {\text{GC}}_{\text{syn}0.8}+{\text{GC}}_{\text{syn}0.8}\times {\text{A}}_{0.3}-{\text{GC}}_{\text{syn}0.3}\times {\text{A}}_{0.3}\\ =\left({\text{A}}_{0.8}-{\text{A}}_{0.3}\right)\times {\text{GC}}_{\text{syn}0.8}+\left({\text{GC}}_{\text{syn}0.8}-{\text{GC}}_{\text{syn}0.3}\right)\times {\text{A}}_{0.3}\\ =\Delta \text{A}\times {\text{GC}}_{\text{syn}0.8}+\Delta {\text{GC}}_{\text{syn}}\times {\text{A}}_{0.3} \end{array}$$

where ΔGC indicates the increased GC-content of an amino acid from low (0.30−0.31) to high-GC (0.79−0.80) regions; GC_syn0.8_ and GC_syn0.3_ are the average GC-content of synonymous codons of an amino acid appearing at the regions with 0.79−0.80 and 0.30−0.31 GC-content, respectively; A_0.8_ and A_0.3_ are the usages of this amino acid in these two regions, respectively. (A_0.8_ − A_0.3_) × GC_syn0.8_ explains the proportion of GC-content changes influencing the changes of amino acid usage. (GC_syn0.8_ − GC_syn0.3_) × A_0.3_ represents the proportion of GC changes influencing the GC changes in the use of high GC-content synonymous codons for that amino acid. It is supposed that the regional GC-content increase would affect the increased usage of an amino acid and its increased GC_syn_. To give a conserved estimate of the contribution, only four high-GC_syn_ amino acids (Ala, Gly, Pro, and Arg) are included in our analysis. We can likewise infer that of an amino acid to the change of AT-content from high (0.79−0.80) to low GC (0.30−0.31) regions.

### Simulation with random sequences

In addition using the real genome sequences, we repeated our analysis on simulated genomes with different level of GC-content as a control. These simulated genomes are composed of random DNA sequences. We used online software to generate random DNA sequences with different GC-content (Villesen 2007), which varies from 0.30 to 0.80 with an equal interval of 0.01, just the same as we described above. In each of these GC-content groups, we generated 10,000 random DNA sequences with different lengths ranging from 1200 to 1500 bp, which is almost the same as the average CDS length of our real sequence data.

### Data availability

All the sequence data are available in public databases as described in *Materials and Methods*.

## Results

### Three types of amino acids based on GC-content of synonymous codons

**Figure 2:**
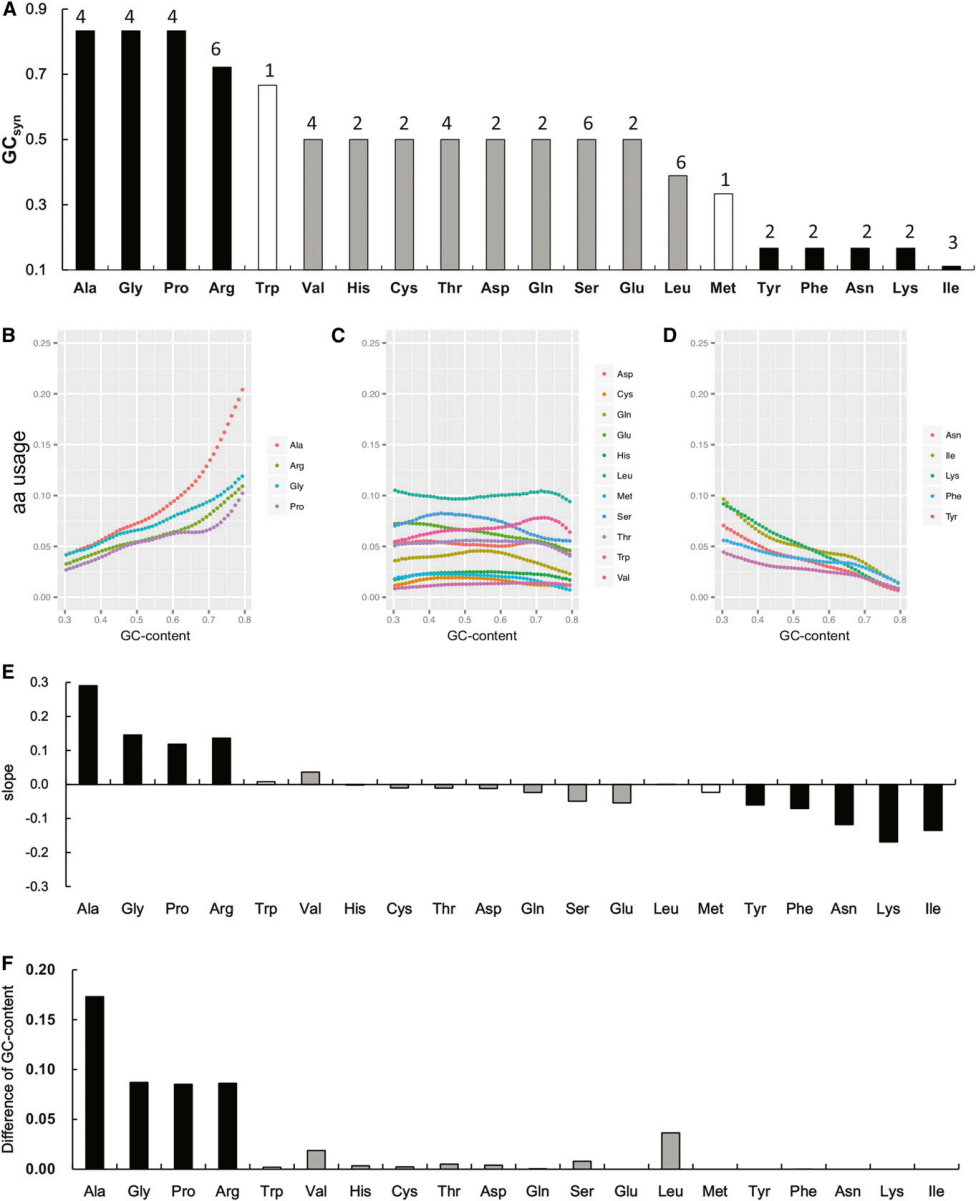
Patterns of amino acid usage and its GC-content of synonymous codons in 65 nuclear genomes in this study. (A) Average GC-content of codons for an amino acid based on the standard DNA genetic code table; the numbers on the bars are the number of synonymous codons for each amino acid; (B−D) Usage frequency variation of amino acids in three groups along with the regional GC-content (in 50 consecutive codons analyze unit), (E) is the slope value in a linear regression analysis for the lines in B−D, and (F) is for the absolute increased GC-content for each amino acid between regions with 0.3−0.31 and 0.79−0.80 GC-content. The black and nonblack bars represent the variably and the less-variably used amino acids, respectively.

We calculated the average GC-content among the synonymous codons for each amino acid (GC_syn_) by using the standard DNA codon table for nuclear genes. GC_syn_ was determined to range from 0.11 to 0.83 (Figure 2A). With respect to the GC_syn_, there are three largely distinct groups of amino acids: four high-, 11 intermediate-, and five low-GC_syn_ amino acids. Within each group, GC_syn_ is the same for most amino acids but lower for one amino acid. Trp and Met are unusual in that they are midway between the high/intermediate and intermediate/low groups, respectively (Figure 2A).

**Figure 3:**
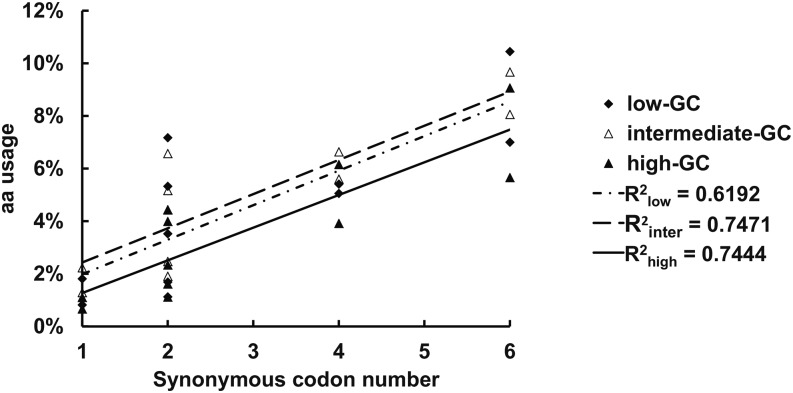
The correlation between amino acid usage and the number of synonymous codons for the 11 less variably used amino acids with intermediate-GC_syn_ at high- (0.79−0.80), intermediate- (0.50−0.51), and low-GC (0.3−0.31) regions.

We used 65 representative genomes from bacteria, plants, and animals to investigate the amino acid usage and codon usage based on GC_syn_ (Supporting Information, Table S1). Our analyses show evidences for a connection between the usage and the number of synonymous codons for each amino acid. First, within each group of amino acid described previously, there is one amino acid (*i.e.*, Arg, Leu, or Ile) for which the GC_syn_ is lower than other members (Figure 2A). Each of these three amino acids has the greatest number of synonymous codons in its group: 6, 6, and 3, respectively. Among these 15 codons are six of the eight least used sense codons (4 for Arg, 1 for Leu, and 1 for Ile) (Table S2). When these less frequently used codons are ignored, each of the three amino acids’ GC_syn_ becomes greater. Second, the single codon for both Trp and Met does not have a GC_syn_ that can fall into one of the three defined groups (Figure 2A), and they represent the least (0.0128, Trp) and the third least-used amino acids (0.0190, Met) in all 65 species (Table S2). Third, there is a positive correlation between amino acid usage and the number of codons for the 11 amino acids in the intermediate-GC_syn_ group, irrespective of the regional GC-content (Figure 3; r = 0.787−0.864, *P* < 0.01). Interestingly, Leu, which is encoded by six synonymous codons, always has the greatest usage (0.100) although the usage frequencies for each of the six individual codons are different. For all 20 amino acids, a significantly positive correlation (*P* < 0.05) is still present in the regions in which GC-content is greater than 0.35 (Table S3). These close connections between GC_syn_, codon usage and the number of synonymous codons suggest possible mechanisms of adaptive evolution.

### Three distinct usage patterns of amino acids

With three obvious types of amino acids based on GC_syn_, one may ask whether this could be related to the different amino acid usage patterns in organisms. To address this question, we focused on the relationship among GC_syn,_ regional GC-content, and amino acid usage in different genomes, hypothesizing that the amino acids with high GC_syn_ could be used more frequently in high-GC regions and contrarily, amino acids with low GC_syn_ could be used more frequently in low-GC regions. In light of this hypothesis, amino acids with intermediate GC_syn_ are expected to be less sensitive to regional GC-content than those with extreme GC_syn_. With the 65 genomes, genome sequences, CDS, and 50-consecutive codons were analyzed separately as a unit from each of these species. We calculated the GC-content and amino acid usage for each unit and classified the results from CDS and 50 consecutive codon analysis units with an interval of 0.01 GC-content from “0.30−0.31” to “0.79−0.80” (see *Materials and Methods* for details; whole-genome comparisons do not result in continuous GC range fractions from one another). Three distinct amino acid usage patterns, consistent with the three types grouped by GC_syn_, were observed regardless of which unit was used (Figure 2, B−D for the 50 consecutive codons unit and Figure S1 for the other two units). The overall usages of the four amino acids with the highest GC_syn_ and the five amino acids with the lowest GC_syn_ both vary with the regional GC-content, whereas the usage of the remaining 11 amino acids with intermediate GC_syn_ is less variable. Of note, this analysis suggests that both Trp and Met fall in with the intermediate group although their GC_syn_ appear midway between adjacent groups (Figure 2A).

To further characterize the three types of amino acids, the slopes of linear regression analyses for the lines in Figure 2, B−D were then calculated to reveal the degree of inclination in response to the increase of GC-content. Interestingly, a steeper incline was always observed in the amino acids with extreme GC_syn_ (Figure 2E). The largest positive slopes (from 0.116 to 0.286; 0.170 on average) were observed in the four amino acids with the greatest GC_syn_ (Ala, Gly, Pro, and Arg; *P* < 0.001 for each regression), whereas the most negative ones (from −0.17 to −0.06; −0.110 on average) appeared in five amino acids with the lowest GC_syn_ (Tyr, Phe, Asn, Ile, and Lys; *P* < 0.001 for each). The other 11 amino acids with intermediate GC_syn_ exhibited relatively flat patterns (Figure 2C) (slope = 0.012 on average; *P* < 0.01). The minor usage changes across regions with different GC-content indicate that these 11 amino acids are less sensitive to the regional GC-content, consistent with our expectation. These results therefore suggest that there is a strong association between GC_syn_ for a given amino acid and its usage variation among regions with different GC-content.

Besides, given that the 65 species we investigated here have very different biology, life history traits, effective populations size, etc., which will greatly affect the selective pressures acting on a genome in general, and on synonymous codon and amino acid usage in particular, we performed the same analysis by separating prokaryotes from eukaryotes. Within eukaryotes, we also separated mammals and plants from other species. Indeed, we still observed the three distinct amino acid usage patterns in these different datasets (Figure S2). Such findings suggest that the correlation is independent of selective pressures and any specific species.

In addition to calculate regional GC-content by considering all the positions in CDS, we also computed GC-content from the third codon positions (GC3-content) because inside a codon, positions 1, 2, and 3 are not under similar evolutionary forces. We computed GC3-content and sorted the sequences based on their GC3-content, which ranged from 0.06 to 0.32, into 26 groups with an equal interval of 0.01. Then we checked the correlation between amino acid usage and GC3-content. We still observed the same three distinct usage patterns of amino acids (Figure S4, A and B). The GC3-content analysis shows that the pattern of amino acid usage and GC3-content is consistent with the results derived from whole GC-content.

### The usages of amino acids and codons are nearly identical for similar GC-content regions

**Figure 4:**
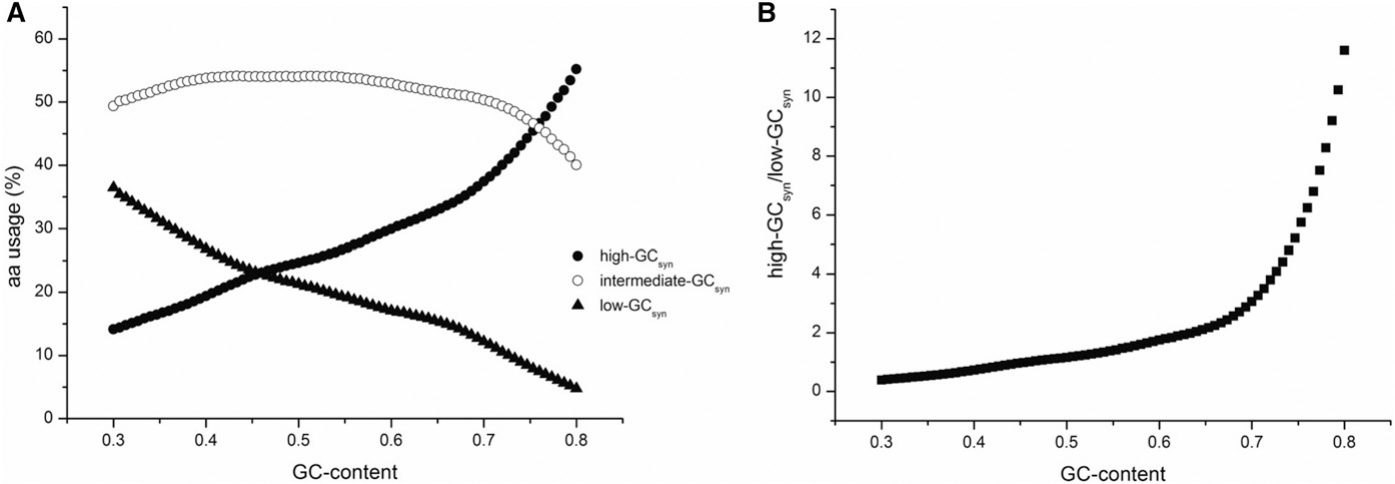
The total usage patterns of three amino acids types. (A) The usage frequencies of high-, low-, and intermediate-GC_syn_ amino acids change when GC-content increase from 0.30 to 0.80. (B) The amino acid usage ratios of the high-GC_syn_ to low-GC_syn_ types are shown.

The regular usage patterns of high-, low- and intermediate- GC_syn_ amino acids (Figure 2, B−D) and the corresponding groups (Figure 4A) suggest their usage is near equilibrium for the given GC-content, regardless of their eukaryotic or prokaryotic origins. When regional GC-content is low, the usage ratio of high- to low-GC_syn_ amino acids is much less than 1, indicating the more frequent uses of low GC_syn_ amino acids. As the GC-content increases, there is a point where high- and low-GC_syn_ amino acids are used equally; after this point the usage ratio of high- to low-GC_syn_ continues to increase gradually (Figure 4B). The dramatic ratio variation reflects the dynamics of amino acid usage and may indicate that it could be associated with functional variations in these motifs. However, in contrast to the high- and low-GC_syn_ groups, the amino acid usage in the intermediate GC_syn_ group shows little variation when GC-content changes from 0.3 to 0.7 (Figure 4A). On the basis of these observations, the usage frequencies would be expected to be the same in different genes or motifs with similar GC-content. We then conducted another linear regression analysis in which we calculated 49 slopes and 49 R^2^s from the 20 pairs of amino acid usage frequency between every two adjacent GC-groups (0.010 GC-difference intervals means every GC nearest pair in Figure 1). Strikingly, both slopes and R^2^s are close to 1 (1.0123 ± 0.025 and 0.9984 ± 0.0006, respectively) when the regional GC-content is similar, strongly suggesting that the usage frequency of a certain amino acid is likely constant in regions with same GC-content.

**Figure 5:**
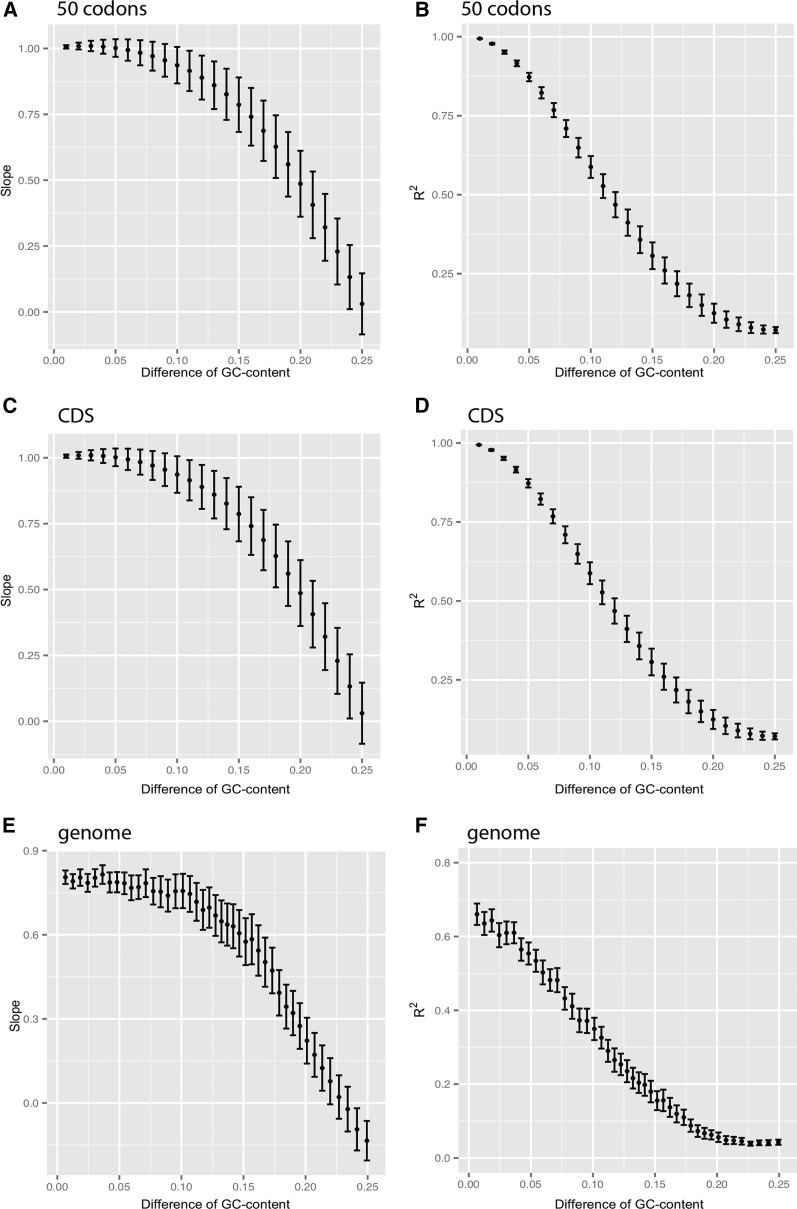
The patterns of the correlation slope (A, C, and E) and the coefficient of determination R^2^ values (B, D, and F) for codon usages between different GC groups. Fifty-codon (A and B), CDS (C and D), and whole genome (E and F) were used respectively as an analysis unit, with a GC-content difference less than 0.25 (the values are nearly 0 when ΔGC > 0.25). As shown in 50 consecutive codons (A and B) and CDS (C and D) analysis units, when the difference of GC-content from two compared units is 0 (x-axis), both the linear regression slope and the regression coefficient R^2^ are infinitely approaching 1 (*P* = 2.32e-54), which strongly suggests that codon usage frequencies are nearly constant in regions with similar GC-content.

The comparable usage of amino acids at similar GC-content may essentially reflect a constant usage of codons. On the basis of the usage of each of the 61 sense codons in the same set of data as previously used for the amino acid usage analysis, regression analysis (Figure 5) reveals that both slopes and R^2^s are still close to 1 (1.0045 ± 0.0737 and 0.9946 ± 0.0038, respectively) when GC-content is similar. As the GC-content difference increases, the observed slope or R^2^ is expected to deviate from 1. To test this expectation, we calculated slope or R^2^ from all possible comparisons between two groups (see *Materials and Methods* for details; Figure 1). Shown in Figure 5, the average slope and R^2^ are reduced with the increase of GC difference between compared groups. For 50 consecutive codons and CDS analysis units, both the slope and R^2^ approach 1 when GC-difference is 0 (Figure 5). In comparison, we used the “genome” analysis unit as a control. The slope and R^2^ are 0.805 and 0.660 respectively in the “genome” unit when comparing two genomes with the smallest GC-content difference (0.0068). The lower values of slope and R^2^ for “genome” than for “50 consecutive codon” and “CDS” units are mainly contributed by the noncoding sequences. Therefore, these results further suggest that compared with the noncoding regions, the usage frequency of each codon (Figure 5) or amino acid (Figure S5) in different genes is more likely to be the same in regions of similar GC-content in the coding regions. In other words, there is a constant codon and amino acid usage when GC-content is similar. We also performed this regression analysis by separating prokaryotes, mammals, plants and other eukaryotes (Figure S3). The results also are consistent, indicating that our results are more generally distributed in living organisms rather than specific for one group. It is also true that when the GC3-content is the same, the R square and slope are both approaching 1 (Figure S4C).

To make our result more convincing, we performed an experiment as a control by repeating the analysis on simulated genomes with different level of GC-content and these simulated genomes are made up of random DNA sequences. We used online software to generate random DNA sequences with different GC-content (Villesen 2007). GC-content varies from 0.30 to 0.80 with an equal interval of 0.01. In each GC-content group, we generated 10,000 random DNA sequences with different lengths ranging from 1200 to 1500 bp. Using these random sequences, we repeated our analysis. Different from what we identified using real sequences, the regression result of random sequences (Figure S6) shows that when GC-content difference is 0.01, the R square is not equal to 1.0 (mean = 0.980, *P* = 0.1316, *t*-test). In comparison, with the real CDS, when GC-content difference is 0.01, the R square is almost 1.0 (mean = 0.994, *P* = 2.008e-12, *t*-test). We also performed a paired *t*-test between the real data and the simulated data, which showed significant differences between each other (*P* < 2.2e-16). This result indicates that when the GC-content is the same, the amino acid usage or codon usage is not constant in random sequences. In addition, we observed a correlation between amino acid usage and GC-content based on different GC_syn_ groups in random sequences as well (Figure S6C). Although the pattern of amino acid usage and GC-content appeared similar between the genomic data and simulated data, we found significant differences between them for the majority of the amino acids (Table S7). This simulation serves as a good control to our analysis of the coding regions and certifies our results: codon usage frequencies are nearly constant in regions with similar GC-content; and that intrinsically harbors some biological significance.

### The influence of GC-content on amino acid composition

Although GC-content is considered widely to determine amino acid usage, it is interesting to quantitatively dissect how that changes the amino acid composition from one region to another. Besides, GC-content also would influence which synonymous codon for a certain amino acid would be used. Either GC-rich or AT-rich synonymous codons would be chosen on the basis of the variation of GC-content. To examine the effects of GC-content, we divided the contributions of GC-content’s effects into two parts: on the use of different amino acids or their synonymous codons that bears different GC fractions. Here we created an equation to quantify these two parameters due to the change of regional GC-content.

$$\Delta \text{GC}=\Delta \text{A}\times {\text{GC}}_{\text{syn}0.8}+\Delta {\text{GC}}_{\text{syn}}\times {\text{A}}_{0.3}$$

ΔGC indicates the increased GC-content of an amino acid from low to high GC-content regions (*e.g.*, GC-content from 0.30−0.31 to 0.79−0.80), which could be split into two components: the amino acid’s increased usage (ΔA) and its increased GC_syn_ (ΔGC_syn_) due to the usage of different synonymous codons. The first component reflects the influence of GC-content variation on amino acid usage. The second component indicates the influence on GC_syn_ change (see details in *Materials and Methods*). Here only the four high-GC_syn_ amino acids are taken into account because these four amino acids account for most of the increased GC-bases from low- to high-GC regions (Figure 2E), and the usage of each increases constantly as the regional GC-content increases (Figure 4A). The total increased GC-content is 0.490 for all the 20 amino acids between the regions with 0.30−0.31 and 0.79−0.80 GC-content. We show that the influence on the use of four high-GC_syn_ amino acids is 0.376, which accounts for 76.7% of the total increased GC-content whereas the influence on synonymous codons accounts for only 8.2% (Figure 2F; Table 1). Similarly, it is also possible to calculate the influence of low-GC_syn_ amino acids to the increase of AT-bases between the regions with 0.79−0.80 and 0.30−0.31 GC-content (Table S4). All these results suggest that high GC-content CDS produces genes with high GC_syn_ amino acids whereas low GC-content sequence encodes genes with low GC_syn_ amino acids. Therefore, GC-content not only determines the codon/amino acid usage but also has great influence on amino acid composition, especially the extreme-GC_syn_ amino acids in a region.

**Table 1:** The effects of GC-content by amino acids from low to high GC regions

Item	GC-Content			aa Usage			GC_syn_			Effects on	
	GC_0.3_	GC_0.8_	ΔGC	A_0.3_	A_0.8_	ΔA	GC_syn0.3_	GC_syn0.8_	ΔGC_syn_	aa	GC_syn_
Ala	0.030	0.199	0.169	0.042	0.204	0.162	0.727	0.973	0.246	0.158	0.010
Gly	0.031	0.114	0.083	0.042	0.119	0.077	0.735	0.954	0.220	0.074	0.009
Pro	0.020	0.098	0.078	0.027	0.102	0.075	0.727	0.963	0.236	0.073	0.006
Arg	0.017	0.103	0.086	0.033	0.109	0.076	0.510	0.943	0.433	0.072	0.014
Total	0.097	0.514	0.416	0.143	0.534	0.391	/	/	/	0.376	0.040
Percentage relative to 0.490					/	/	/	/	/	76.7%	8.2%

## Discussion

All of our findings initiated from a simple parameter, the GC-content of all the synonymous codons for an amino acid (GC_syn_). On the basis of GC_syn_, our results show that the 20 amino acids fall into three distinct groups. The usage of amino acids from different groups is distinct from each other. This result further led us to find constant amino acid usage when GC-content is determined. Therefore, GC-content reveals the profound influence on genomic architecture and divergence. In this point of view, it will shed light on our understanding of fundamental biological questions.

Genetic codons have long been considered to bear no inherent advantages (Francino and Ochman 1999; Yang and Nielsen 2008) and resulted from a “frozen accident” (Crick 1968). Codon reassignment could change the amino acid sequences of most proteins, resulting in a destructive impact on the organism. However, these ideas are inconsistent with the patterns observed in this report. The association between usage and synonymous codon numbers of amino acids with different GC_syn_ suggests that a specific codon reassignment could be associated with the usage variation of both the codon and amino acid. In this light, the least used codons presumably have a greater potential to be reassigned because this type of coding changes would have the least impact on the genome. Indeed, several lines of evidence support this point. First, the majority of nonuniversal codons in mitochondria genomes, ATA, AGA, AGG, and CTA (Li and Tzagoloff 1979; Barrell *et al.* 1979; Andersson and Kurland 1991; Knight *et al.* 2001), include four of the eight least used codons (Table S2) in our analyzed dataset. These four codons encode Ile, Arg, and Leu, which are exactly the same amino acids with the lowest GC_syn_ in each distinct GC_syn_ group. Second, Trp and Met that are each encoded by a single codon have an unusual GC_syn_, and are used two times more frequently in mitochondrial genomes than in nucleic genomes (Table S5). Third, the bacterial *Mycoplasma* species translate UGA as Trp, which is a property also observed in animal mitochondria. Trp is among the least used amino acids and possesses a GC_syn_ different from other amino acids. These evidences confirm that GC_syn_-based association has a great influence on the reassignment of specific codons and, subsequently, the encoded amino acids.

Besides the influence on reassignment, the association between usage pattern and synonymous codon numbers of amino acids with different GC_syn_ also may suggest an adaptive pattern in the course of evolution. For example, amino acids (Arg, Leu, or Ile) with a relatively low GC_syn_ in each group also have the most synonymous codons within that group, some of which are the least used codons. If these atypically used codon(s) are ignored, each of the three GC_syn_ becomes greater and is more similar to others in the corresponding group. Trp and Met both have unique GC_syn_ and also are used infrequently, suggesting that the single codon may only fit to the least-used amino acids because they do not have synonymous codons and consequently their GC_syn_ are fixed across genomic regions. In addition to these five amino acids, the usage of the eight less-variable amino acids with GC_syn_ of 0.5 is positively correlated with their codon numbers, which also provides evidence for adaptive evolution.

The most striking finding in our study is constant codon and amino acid usage in the regions with similar GC-content even though the organisms selected for genome comparisons in our study are widely represented. In other words, this conclusion is beyond the phylogenetic constraints and demonstrates a unique spectrum of amino acid usage that applies to all species, which is extremely critical to understanding amino acid usage and nucleotide sequences in poorly characterized taxa. In addition to this implication, this observation also can lead to an assumption that the fitness of the organism would be greater if the usage of codons and amino acids is in concordance with underlying regulations at the level of GC-content. A recent study in *Escherichia coli* using *GFP* genes varying in GC-content directly supports our assumption. In that study, *E coli* strains harboring GC-rich versions of *GFP* genes display a greater growth rate (Raghavan *et al.* 2012). We further analyzed their experimental data and were interested to discover that the strains showing a greater growth rate are the ones harboring *GFP* genes with a similar GC-content to that in the *E. coli*’s overall CDS (Table S6). The more GC-content in *GFP* deviated from that in intrinsic CDSs (0.519), the lower the growth rate displayed. The gene harboring the same GC-content as the genomic CDS might give rise to more efficient/accurate transcription and translation, resulting in greater fitness. The authors explain their result as a selective force driving genes toward greater GC-content, which is contrary to the pervasive mutational bias toward AT (Raghavan *et al.* 2012). We suggest an alternative hypothesis in which selection is strong when codon bias levels deviate from the equilibrium level but weak when it reaches equilibrium. Additional experiments are required to test our equilibrium model.

Another critical question we want to address is whether the GC-content variation across the genome is due to intrinsic mechanisms or external factors, and whether it is a result of a neutral process or selection (Eyre-Walker and Hurst 2001). As is well known, the genomic organization of GC-rich regions differs significantly from that of GC-poor regions, including gene densities (Lander *et al.* 2001), CpG island densities (Cross *et al.* 2000), and repetitive DNA elements densities (Bernardi 2000). Genes in GC-rich regions also have been argued to yield different proteins from those in GC-poor regions (D’Onofrio *et al.* 1991), and genes in GC-rich regions generally were found to have increased expression levels especially in mammalian cells. We provide a different approach to explain those findings. Using a formula to measure the influence of regional GC-content change on amino acid usage and GC_syn_ separately and quantitatively, we show in our results that GC-content produces the organization of high-, low-, and intermediate- GC_syn_ amino acids, particularly those with extreme GC_syn_ in any region. This may be related with gene functions. Tatarinova *et al.* (2010) demonstrated that GC-content is greater for genes from electron transport or energy pathways, response to abiotic or biotic stimuli, response to stress, transcription and signal transduction. Accompanying the requirement for GC-content variation, the synonymous codons have to be changed toward a higher (or lower) GC state. The usage changes in the amino acids by the gene’s GC-content also can facilitate and perhaps modulate the formation of genomic structures. Our study further suggests why a particular synonymous codon may be preferentially used. Due to functional requirements, the usage of amino acids in genes could be predetermined. Given that, the different GC-content of synonymous codons makes it possible to adjust more efficiently to the regional GC changes. A close correlation was previously revealed between exon GC-content and its surrounding intron GC-composition (Zhu *et al.* 2009). Our observation of functionally associated exon GC-composition provides good evidence for the influence of GC-content from exons to introns, and it will greatly further our understanding of the evolution of gene architecture.

## 
